# Contextuality with Disturbance and without: Neither Can Violate Substantive Requirements the Other Satisfies

**DOI:** 10.3390/e25040581

**Published:** 2023-03-28

**Authors:** Ehtibar N. Dzhafarov, Janne V. Kujala

**Affiliations:** 1 Department of Psychological Sciences, Purdue University, West Lafayette, IN 47907, USA; 2 Department of Mathematics and Statistics, University of Turku, FI-20014 Turun yliopisto, Finland

**Keywords:** contextual equivalence, contextuality, consistent connectedness, consistification, connections, disturbance, signaling

## Abstract

Contextuality was originally defined only for consistently connected systems of random variables (those without disturbance/signaling). Contextuality-by-Default theory (CbD) offers an extension of the notion of contextuality to inconsistently connected systems (those with disturbance) by defining it in terms of the systems’ couplings subject to certain constraints. Such extensions are sometimes met with skepticism. We pose the question of whether it is possible to develop a set of substantive requirements (i.e., those addressing a notion itself rather than its presentation form) such that (1) for any consistently connected system, these requirements are satisfied, but (2) they are violated for some inconsistently connected systems. We show that no such set of requirements is possible, not only for CbD but for all possible CbD-like extensions of contextuality. This follows from the fact that any extended contextuality theory T is contextually equivalent to a theory $T^{\prime }$ in which all systems are consistently connected. The contextual equivalence means the following: there is a bijective correspondence between the systems in T and $T^{\prime }$ such that the corresponding systems in T and $T^{\prime }$ are, in a well-defined sense, mere reformulations of each other, and they are contextual or noncontextual together.

## 1. Introduction

A formal theory T of contextuality is defined by a class R of possible systems of random variables and a rule by which these systems are divided into noncontextual and contextual ones. In the original theory of contextuality (a term in which we include both the Kochen–Specker contextuality and the contextuality in distributed systems, referred to as nonlocality [1,2,3,4,5,6,7,8]), the class R is confined to consistently connected systems, or a subclass thereof. These are the systems with no “disturbance” or “signaling,” which means that the variables representing the same property (answering the same question) in different contexts are identically distributed. The Contextuality-by-Default theory (CbD) extends the notion of contextuality to all systems of random variables, including those with disturbance [9,10], and it has been applied to several experimental and theoretical situations [11,12,13,14,15,16,17,18]. A recent workshop on contextuality [19] exhibited a renewed interest to studying contextuality in inconsistently connected systems, including approaches that are distinctly non-CbD-like [20,21,22], and some work directly critical of CbD ([23], responded to in Ref. [24]).

The present paper is not about CbD specifically. Rather, it is about a broad class of all possible *CbD-like theories*, as defined below. The plan and the main message of the paper are as follows. In Section 2, we present the terminology and notation to be used and define the notion of a system of random variables modeling (representing or describing) another system. In Section 3, we define the traditional notion of contextuality in the language of probabilistic couplings [25], and we introduce the notion of C-contextuality as a very broad generalization of both traditional contextuality and CbD-contextuality. In Section 4, we introduce the notion of consistification of a system and show that any theory T, irrespective of its class R of systems and a specific version of the C-contextuality it uses, can be redefined as a theory $T^{\prime }$, whose systems are consistently connected, and that uses the traditional notion of contextuality. Because of this, we conclude that there can be no set of substantive requirements X for the notion of contextuality that are satisfied by all consistently connected systems but contravened by some inconsistently connected ones. Indeed, if such a set of requirements existed, one could form a theory T whose class R includes some systems contravening X. However, X would then be satisfied by the theory $T^{\prime }$ that is contextually equivalent to T and a mere reformulation thereof. Consequently, requirements X cannot be substantive: they address a form rather than the substance of the notion of contextuality. In Section 5, we discuss some issues related to the consistified systems (the term used for the consistently connected systems in $T^{\prime }$), including the representability thereof by hidden variable models. We also briefly discuss there a still more general (in fact, maximally general) notion of C-contextuality, one that does not have the existence-and-uniqueness property postulated for C-contextuality. In the final analysis, this does not alter the main conclusion of the paper.

The idea that consistification precludes the possibility of rejecting extended contextuality while accepting the traditional one was previously mentioned in Ref. [24]. However, it was confined to CbD only and mentioned without elaborating. The consistification procedure was first described in Ref. [13] for an older version of the CbD approach, and it was elaborated and adapted to the current version of CbD in Ref. [26]. Finally, the C-contextuality in our paper generalizes a more limited version of C-contextuality that was used in Ref. [27] as a generalization of the CbD approach.

## 2. Basic Notions

A *system* of random variables is a set of double-indexed random variables

(1) $$R=\left\{R_{q}^{c}:c\in C,q\in Q,q≺c\right\},$$

where q∈Q identifies what the random variable $R_{q}^{c}$ represents (measures, responds to, or describes); c∈C identifies circumstances under which $R_{q}^{c}$ is recorded (including what other random variables are recorded together with $R_{q}^{c}$); *q* and *c* are referred to as, respectively, the *content* and the *context* of the random variable $R_{q}^{c}$; and the relation q≺c indicates that a variable with content *q* is recorded in context *c*. As an example, this is a system with $Q=\left\{1,2,3\right\}$ and $C=\left\{1,2,3,4\right\}$:
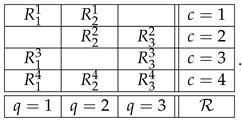
(2)

The subset $R^{c}=\left\{R_{q}^{c}:q\in Q,q≺c\right\}$ of random variables recorded in the same context *c* (a row in the matrix above) is termed a *bunch*, and the subset $R_{q}=\left\{R_{q}^{c}:c\in C,q≺c\right\}$ of random variables sharing a content *q* (a column in the matrix above) is termed a *connection*. The difference in font ($R^{c}$ vs. $R_{q}$) reflects the fact that $R^{c}$ is a random variable in its own right (i.e., all its components are jointly distributed), whereas the components of $R_{q}$ are not jointly distributed. In fact, no two random variables $R_{q}^{c}$ and $R_{q^{\prime }}^{c^{\prime }}$ are jointly distributed unless they are in the same bunch, $c=c^{\prime }$. The measurable space on which $R_{q}^{c}$ is distributed is assumed to be the same for all elements of a connection and can be denoted $\left(A_{q},\Sigma_{q}\right)$.

The triple $\left(Q,C,≺\right)$ is called the *format* of the system. It is essentially the mathematical depiction of “what the system is about,” what kind of empirical or theoretical situation it represents. Thus, the format of the system in (2) can be presented as

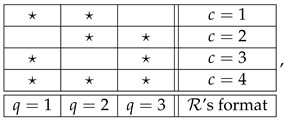
(3)
where 🟉 indicates the elements of the relation ≺. To define a system of a given format, one has to specify the distributions of its bunches.

As should be clear from Abstract and Introduction, in this paper, we use the notion of one system of random variables, B, being a “mere reformulation” of another, A. Intuitively, this means that regardless of what empirical or theoretical situation is modeled (described, represented) by A, it is also modeled by B. The relation between a system and a situation it depicts is difficult to formalize directly, as one would have then to impose some formal structure on the situation being represented before it is represented (as in the representational theory of measurement, [28,29]). However, it is sufficient for our purposes to formalize a simpler relationship: between a system A and another system that models (describes, represents) the system A. Moreover, rather than presenting this relationship in a most general possible way, it will suffice to describe one special, universally applicable construction of the modeling systems B. We will refer to this construction as *canonical modeling*.

Consider two classes of systems, R and $R^{†}$, in a bijective correspondence to each other, about which we say that any system in R is canonically modeled by the corresponding system in $R^{†}$. The following definition gives a precise meaning to this relation.

**Definition** **1.** *We say that a system R∈R with format $\left(Q,C,≺\right)$ is canonically modeled by a system $R^{†}\in R^{†}$ with format $\left(Q^{†},C^{†},{≺}^{†}\right)$ if*

**(canonical contents)**  *$Q^{†}=\left\{\left(q,c\right):q≺c\right\}$,*
**(canonical contexts)**  *$C^{†}=\left\{\left(\cdot ,c\right):c\in C\right\}⊔\left\{\left(q,\cdot \right):q\in Q\right\}$,*
**(canonical relation)**  *$\left(q,c\right){≺}^{†}\left(\cdot ,c\right)⟺\left(q,c\right)\in Q^{†}$, and $\left(q,c\right){≺}^{†}\left(q,\cdot \right)⟺\left(q,c\right)\in Q^{†},$*
**(main bunches)** 
$R^{\left(\cdot ,c\right)}=\left\{R_{\left(q,c\right)}^{\left(\cdot ,c\right)}:\left(q,c\right){≺}^{†}\left(\cdot ,c\right)\right\}\overset{d}{=}\left\{R_{q}^{c}:q≺c\right\}=R^{c}$

**(auxiliary bunches)**  *$R^{\left(q,\cdot \right)}=\left\{R_{\left(q,c\right)}^{\left(q,\cdot \right)}:\left(q,c\right){≺}^{†}\left(q,\cdot \right)\right\}$ is uniquely determined by the distributions of the corresponding variables in $R_{\left(q,\cdot \right)}=\left\{R_{\left(q,c\right)}^{\left(\cdot ,c\right)}:\left(q,c\right){≺}^{†}\left(q,\cdot \right)\right\}$.*

Here, the symbol $\overset{d}{=}$ stands for “has the same distribution as”. The dot symbol in $\left(\cdot ,c\right)$ and $\left(q,\cdot \right)$ should be taken as part of the names of these contexts. We choose this notation to emphasize that every random variable $R_{q}^{c}$ of the system R is placed in $R^{†}$ within two contexts, $\left(\cdot ,c\right)$ and $\left(q,\cdot \right)$, whose names are derived from the indices of the variable. Note that the variables in the set $R_{\left(q,\cdot \right)}$ defined here have the same distributions as the corresponding variables in $R_{q}=\left\{R_{q}^{c}:q≺c\right\}$. We use the former set, however, to emphasize that the auxiliary bunches are uniquely determined by the corresponding variables in the main bunches. Note that the variables in $R_{\left(q,\cdot \right)}$ are not jointly distributed, so $R^{\left(q,\cdot \right)}$ depends on their individual distributions only.

To give an example, consider the systems

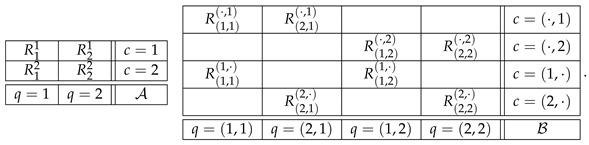
(4)
Observe that in system B the contents, contexts, and the relation between them are constructed in accordance with Definition 1. System B canonically models system A if

(5) $$\left\{R_{\left(1,1\right)}^{\left(\cdot ,1\right)},R_{\left(2,1\right)}^{\left(\cdot ,1\right)}\right\}\overset{d}{=}\left\{R_{1}^{1},R_{2}^{1}\right\},\;\left\{R_{\left(1,2\right)}^{\left(\cdot ,2\right)},R_{\left(2,2\right)}^{\left(\cdot ,2\right)}\right\}\overset{d}{=}\left\{R_{1}^{2},R_{2}^{2}\right\},$$

and if there is a rule by which the distribution of

(6) $$R^{\left(q,\cdot \right)}=\left\{R_{\left(q,1\right)}^{\left(q,\cdot \right)},R_{\left(q,2\right)}^{\left(q,\cdot \right)}\right\},\;q=1,2,$$

is uniquely determined by the distributions of the corresponding variables in

(7) $$R_{\left(q,\cdot \right)}=\left\{R_{\left(q,1\right)}^{\left(\cdot ,1\right)},R_{\left(q,2\right)}^{\left(\cdot ,2\right)}\right\},\;q=1,2.$$

Observe the following properties of canonical modeling.

1. The formats of R and $R^{†}$ are reconstructible from each other, and so are the bunches of the two systems. Moreover, $R^{†}$ faithfully replicates the bunches of R. This allows one to say that R and $R^{†}$ describe the same empirical or theoretical situation.
2. One might wonder why we need the auxiliary contexts at all, and they are indeed unnecessary if all one wants is a system modeling another system, e.g.,

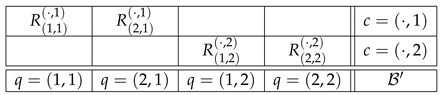
  However, we will see the utility of the auxiliary contexts when we introduce consistifications and contextual equivalence, in Section 4.
3. The contents in the modeling system are “contextualized”. For instance, system A in (4) may be describing an experiment in which two questions, q=1 and q=2, are asked in two orders, c=1 indicating “1 then 2” and c=2 indicating “2 then 1” [30,31]. In this case, in the modeling system, the content $q=\left(1,2\right)$ should be interpreted as “question 1 asked second”, and $q=\left(1,1\right)$ should be interpreted as “question 1 asked first”. We return to the issue of interpretation in Section 5.1.
4. The indexation of the variables in a canonical model is clearly redundant, and it can be simplified. It is more important, however, to maintain the general logic of indexing the variables by their contents and contexts.

## 3. Traditional and Extended Contextuality

A system R is *consistently connected* if in every connection $R_{q}$ all its constituent variables have one and the same distribution. Otherwise, the system is *inconsistently connected*. (The latter term is also used to designate arbitrary systems, i.e., in the meaning of “not necessarily consistently connected”.)

An *overall coupling* of a system R in (1) is an identically labeled system

(8) $$S=\left\{S_{q}^{c}:c\in C,q\in Q,q≺c\right\}$$

of *jointly distributed* random variables such that its bunches $S^{c}$ are distributed as the corresponding bunches $R^{c}$,

(9) $$S^{c}\overset{d}{=}R^{c}.$$

Clearly, *S* has the same format as R. A *coupling* $S_{q}$ of a connection $R_{q}$ is a set

(10) $$S_{q}=\left\{S_{q}^{c}:c\in C,q≺c\right\}$$

of jointly distributed random variables such that $S_{q}^{c}\overset{d}{=}R_{q}^{c}$ for all its elements. A connection coupling $S_{q}$ is said to be an *identity coupling* if $S_{q}^{c}=S_{q}^{c^{\prime }}$ for any two of its elements. Obviously, such a coupling exists if and only if all of its elements (equivalently, all elements of the connection $R_{q}$) have one and the same distribution. Moreover, the identity coupling is unique if it exists. (The uniqueness of a coupling should always be understood as the uniqueness of its distribution. In other words, it is irrelevant on what domain probability space the coupling is defined as a random variable.)

The traditional notion of contextuality is confined to consistently connected systems, and it can be rigorously defined in our terminology as follows.

**Definition** **2.** *A consistently connected system R∈R is noncontextual if it has a coupling S in which any connection $S_{q}$ is the identity coupling of the connection $R_{q}$. Otherwise, the system is contextual.*

The class of all possible systems R in a theory T is denoted R. For instance, R can only contain the systems with finite sets *Q* and *C*, or only the systems with dichotomous random variables. By constraining the class R, one induces constraints on all possible random variables, $R_{q}^{c}\in R_{+}^{+}$, on bunches of random variables, $R^{c}\in R^{+}$, and on possible connections, $R_{q}\in R_{+}$.

In CbD, contextuality of a system R is defined by considering its couplings *S* and determining if, in some of them, the couplings $S_{q}$ of the system’s connections $R_{q}$ satisfy a certain statement. To generalize this definition to all possible CbD-like theories, all one has to do is to replace this specific statement with one that is (almost) arbitrary. Let C be any statement of the form “the coupling of connection $R_{q}$ has the following properties: …”. The only constraints we impose on C are as follows.

**Definition** **3.** *C is considered well-fitting if (1) for any connection $R_{q}\in R_{+}$, there is one and only one coupling $S_{q}$ of $R_{q}$ that satisfies C, and (2) if $R_{q}$ consists of identically distributed random variables, then the coupling that satisfies C is the identity coupling. We denote such a coupling of $R_{q}$ as $C\left[R_{q}\right]$.*

To give an example of a well-fitting statement C: in CbD, if the class R of all possible systems is confined to the systems with dichotomous variables, the well-fitting statement is C= “for any two random variables $S_{q}^{c_{1}}$ and $S_{q}^{c_{2}}$ in the coupling of connection $R_{q}$, the probability of $S_{q}^{c_{1}}=S_{q}^{c_{2}}$ is maximal possible”. Another example: if the class R of all possible systems is confined to the systems with real-valued (or more generally, linearly ordered) variables, then a well-fitting statement can be C= “for any two random variables $S_{q}^{c_{1}}$ and $S_{q}^{c_{2}}$ in the coupling of connection $R_{q}$, $S_{q}^{c_{1}}$ and $S_{q}^{c_{2}}$ have the same quantile rank”. In Section 5.3, we discuss the possibility of dropping the first of the two defining properties of a well-fitting statement C.

**Definition** **4.** *Given a well-fitting C, a system R is C-noncontextual if it has a coupling S such that, for any connection $R_{q}$ of the system, the connection coupling $S_{q}$ coincides with $C\left[R_{q}\right]$. Otherwise, the system is C-contextual.*

## 4. Equivalence and Impossibility Theorems

It follows from the last two definitions that, for a well-fitting C, a consistently connected system is C-noncontextual if and only if it is noncontextual in the traditional sense (i.e., in the sense of Definition 2). In other words, any extension of the notion of contextuality using a well-fitting C properly reduces to the traditional notion when confined to consistently connected systems. This is not, obviously, sufficient to consider the extension of contextuality by means of a well-constructed C. There may be other desiderata for a well-constructed notion of contextuality, and a specific choice of C may not satisfy them. The question we pose now is as follows:

Q*: Is it possible to formulate a set of such desiderata/requirements X for the notion of contextuality that, for some choice of C, (1) X is satisfied for any consistently connected system, but (2) X is not satisfied for some inconsistently connected systems?

Note that we impose no constraints on what X may entail, except for its being related to contextuality. It may, e.g., for some relation *B* between systems, have the form “if system $R_{1}$ is (non)contextual, then any system $R_{2}$ related to $R_{1}$ by *B* is (non)contextual” [24].

To answer the question Q*, we need the following result.

**Theorem** **1.** *For any well-fitting* C *and system R, there is a consistently connected system $R^{‡}$ that canonically models it (Definition 1), such that R is *C*-contextual (Definition 4) if and only if $R^{‡}$ is contextual in the traditional sense (Definition 2).*

**Proof.**  Let $R^{‡}$ be a canonically modeling system for R, with

$$R^{\left(q,\cdot \right)}=\left\{R_{\left(q,c\right)}^{\left(q,\cdot \right)}:\left(q,c\right){≺}^{‡}\left(q,\cdot \right)\right\}\overset{d}{=}C\left[\left\{R_{q}^{c}:q≺c\right\}\right]=C\left[R_{q}\right].\;\;\;\;\left(*\right)$$

One can check that $R^{‡}$ is consistently connected: every connection $R_{\left(q,c\right)}$ of $R^{‡}$ consists of precisely two variables, $R_{\left(q,c\right)}^{\left(\cdot ,c\right)}$ and $R_{\left(q,c\right)}^{\left(q,\cdot \right)}$, where $R_{\left(q,c\right)}^{\left(\cdot ,c\right)}\overset{d}{=}R_{\left(q,c\right)}^{\left(q,\cdot \right)}$. Indeed, $R_{\left(q,c\right)}^{\left(\cdot ,c\right)}\overset{d}{=}R_{q}^{c}$, because $R^{\left(\cdot ,c\right)}\overset{d}{=}R^{c}$ in any canonically modeling system and $R_{\left(q,c\right)}^{\left(q,\cdot \right)}\overset{d}{=}R_{q}^{c}$ because we know from (*) that $R_{\left(q,c\right)}^{\left(q,\cdot \right)}\overset{d}{=}S_{q}^{c}$, where $S_{q}^{c}\in C\left[R_{q}\right]$.

The system $R^{‡}$ thus constructed is referred to as a *consistification* of R. We can now define the consistification $S^{‡}$ of a coupling *S* of a system in precisely the same way as for the system itself, except that (*) is replaced with the straightforward

$$S^{\left(q,\cdot \right)}=S_{q},$$

with the obvious correspondence between the different indexations within the two random vectors. Clearly, $S^{‡}$ is a coupling of $R^{‡}$.

Assume now that R is noncontextual. This means that it has a coupling *S* such that (a) $S^{c}\overset{d}{=}R^{c}$ for every c∈C and (b) $S_{q}=C\left[R_{q}\right]$ for every q∈Q. Then, in the coupling $S^{‡}$ of system $R^{‡}$, we have (a’) $S^{\left(\cdot ,c\right)}\overset{d}{=}R^{\left(\cdot ,c\right)}$ for every $\left(\cdot ,c\right)\in C^{‡}$, and (b’) $S^{\left(q,\cdot \right)}=C\left[R_{q}\right]$ for every $\left(q,\cdot \right)\in C^{‡}$. Moreover, since both $S_{\left(q,c\right)}^{\left(\cdot ,c\right)}$ and $S_{\left(q,c\right)}^{\left(q,\cdot \right)}$ equal $S_{q}^{c}$, we have (c’) $S_{\left(q,c\right)}^{\left(\cdot ,c\right)}=S_{\left(q,c\right)}^{\left(q,\cdot \right)}$. However, (a’)-(b’)-(c’) mean that $R^{‡}$ is noncontextual in the traditional sense. The implication here is easily seen to be reversible, and we conclude that R is noncontextual if and only if so is $R^{‡}$. □

In our example (4), B is a consistification of A if we specify the rule for the auxiliary bunches as follows: $R_{\left(q,c\right)}^{\left(q,\cdot \right)}\overset{d}{=}R_{\left(q,c\right)}^{\left(\cdot ,c\right)}$, and the distribution of $R^{\left(q,\cdot \right)}$ is the same as that of $C\left[R_{q}\right]$. If C is chosen as in CbD, the consistification of the system R in (2) is the system below (omitting, for simplicity, the parentheses and commas in $R_{\left(q,c\right)}^{\left(\cdot ,c\right)}$ and $R_{\left(q,c\right)}^{\left(q,\cdot \right)}$):
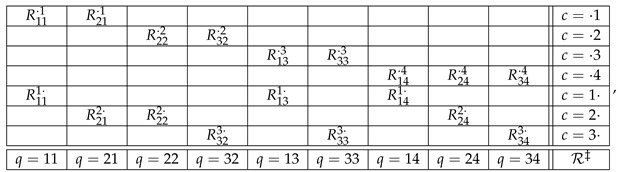
(11)
where all variables are assumed to be dichotomous, and in each of the auxiliary bunches, the variables are pairwise equal with maximal possible probability.

For the purposes of contextuality analysis, $R^{‡}$ can be viewed as a mere reformulation of R, a different form of the same substance. We express this fact by saying that R and $R^{‡}$ are *contextually equivalent*. (In Refs. [24,26], contextual equivalence is defined more narrowly, requiring also the numerical coincidence of certain measures of contextuality, such as contextual fraction [32]. In this paper, however, the level of abstraction is higher, and we only consider the notion of contextuality rather than its quantifications.)

Consider now a theory of (generally, extended) contextuality $T=T\left(C,R\right)$. In accordance with Theorem 1, we can form the class $R^{‡}$ of the consistifications of the elements of R in a bijective correspondence with R. By extension of the term, we can say that T and $T^{\prime }=T\left(C_{0},R^{‡}\right)$ are *contextually equivalent*. $C_{0}$ here denotes the statement “the connection $R_{q}$ has an identity coupling” that underlies the traditional notion of contextuality, because by definition, it can be viewed as a special case of any well-fitting statement C. We have now everything we need to demonstrate our main conclusion. Let there be a set of requirements X of the notion of contextuality that are satisfied by all consistently connected systems (using the traditional contextuality) and contravened by some inconsistently connected ones, using some version of C-contextuality. Let T include some of the inconsistently connected systems contravening X. Clearly then, requirements X contradict theory T, but they are satisfied by the contextually equivalent theory $T^{\prime }=T\left(C_{0},R^{‡}\right)$. Therefore, X is not a set of substantive requirements. We can summarize this as a formal theorem.

**Theorem** **2.** *For any well-fitting* C*, there can be no set of substantive requirements X of the notion of contextuality that are satisfied by all consistently connected systems (using the traditional contextuality) and contravened by some inconsistently connected ones, using C-contextuality.*

Of course, a set of requirements X satisfied by $T^{\prime }$ but not T can be readily formulated. The theorem says, however, that all it can do is lead one to prefer one of two equivalent representations of contextuality, without affecting the substance of the notion.

Note also that in the theorem just formulated, we assume no relationship between the set of requirements X and the bijective correspondence relating R to $R^{‡}$. In particular, let X have the form “if system $R_{1}$ is contextual, then any system $R_{2}$ related to $R_{1}$ by relation *B* is contextual.” It is not necessary then, although not excluded either, that $R_{2}^{‡}$ is also related to $R_{1}^{‡}$ by relation *B*. All that is stated in the theorem above is that if one wishes to use this X as a substantive principle in testing competing theories, then the failure of a theory to satisfy it cannot be selectively attributed to the fact that its R contains inconsistently connected systems.

## 5. Miscellaneous Remarks

Here, we consider a few issues related to the main point of this paper.

### 5.1. Interpretation of Contents and Contexts

Dealing with consistified systems $R^{‡}$, one needs to get used to a new interpretation of contents and contexts of the random variables: as mentioned previously, in $R^{‡}$, contents are “contextualized,” with $\left(q,c\right)$ in place of just *q*, and the contexts are simply marginalized contents, $\left(\cdot ,c\right)$ and $\left(q,\cdot \right)$. Consider as an example the EPR/Bohm experiment, the most widely investigated paradigm in contextuality/nonlocality research [1,33,34]. In the usual CbD notation, the system representing it is

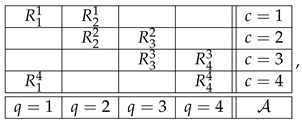
(12)
where q=1 and q=3 denote two settings (axes) to be chosen between by Alice, q=2 and q=4 are settings to choose between by Bob, *c* indicates the combination of their choices, and $R_{q}^{c}$ is the dichotomous (spin-up/spin-down) variables. The consistified representation of the same experiment is (again, omitting the parentheses and commas in the indexation)

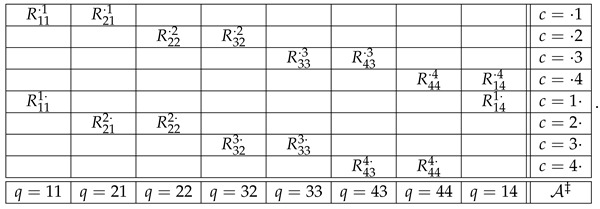
(13)
The interpretation of, say, the content $q=\left(3,2\right)$ here is as follows: it is the choice of axis 3 (that we know to be made by Alice) when Bob’s choice of his axis forms combination 2 with Alice’s choice (which we know to mean that Bob chooses axis 2). The interpretation of context $c=\left(\cdot ,2\right)$ is that it is simply the set of contents whose second component is 2. Similarly, $c=\left(3,\cdot \right)$ is the set of contents whose first component is 3. The random variables within context $c=\left(\cdot ,2\right)$ are jointly distributed by observation, whereas the random variables within context $c=\left(3,\cdot \right)$ are jointly distributed by computation (that, in turn, is uniquely determined by the observations). If C is defined in accordance with CbD, $\left(R_{21}^{2\cdot },R_{22}^{2\cdot }\right)$ is computed so that $R_{21}^{2\cdot }\overset{d}{=}R_{21}^{\cdot 1}$, $R_{22}^{2\cdot }\overset{d}{=}R_{22}^{\cdot 2}$ (consistent connectedness), and the probability of $R_{21}^{2\cdot }=R_{22}^{2\cdot }$ is maximal possible. In particular, if $R_{21}^{\cdot 1}\overset{d}{=}R_{22}^{\cdot 2}$, then $R_{21}^{2\cdot }=R_{22}^{2\cdot }$.

### 5.2. Hidden Variable Models

One possible argument against contextuality in inconsistently connected systems is that it is not distinguishable from inconsistent connectedness itself in the language of hidden variable models (HVMs). If, the argument goes, a consistently connected system R in (1) is noncontextual, it has a coupling *S* in which all random variables can be presented as

(14) $$S_{q}^{c}=F\left(q,\Lambda \right),$$

where Λ is a “hidden” random variable [35]. If R is contextual, then all its couplings can only be presented as

(15) $$S_{q}^{c}=F\left(q,c,\Lambda \right),$$

with ineliminable *c*. However, the latter HVM representation is also required for all inconsistently connected systems, irrespective of whether they are C-contextual or C-noncontextual. We would argue in response that this only means that on this general level (merely showing the arguments of the functions), the language of HVMs is too crude to capture the subtler properties of the couplings, such as contextuality under inconsistent connectedness. However, even if one takes this issue as a matter of concern, it is eliminated by the consistification procedure. The system $R^{‡}$ corresponding to R is noncontextual if and only if it has a coupling $S^{‡}$ such that, for all $\left(q,c\right)\in Q^{‡},$

(16) $$S_{\left(q,c\right)}^{\left(\cdot ,c\right)}=G\left(\left(q,c\right),\Lambda \right)=S_{\left(q,c\right)}^{\left(q,\cdot \right)},$$

for some random variable Λ. If $R^{‡}$ is contextual, then in all its couplings, for some $\left(q,c\right)\in Q^{‡},$$S_{\left(q,c\right)}^{\left(\cdot ,c\right)}\neq S_{\left(q,c\right)}^{\left(q,\cdot \right)}$, which means that their HVM representations can only be different functions,

(17) $$S_{\left(q,c\right)}^{\left(\cdot ,c\right)}=G_{1}\left(\left(q,c\right),\Lambda \right),\;S_{\left(q,c\right)}^{\left(q,\cdot \right)}=G_{2}\left(\left(q,c\right),\Lambda \right),$$

or, equivalently, the same function but with differently distributed hidden variables,

(18) $$S_{\left(q,c\right)}^{\left(\cdot ,c\right)}=H\left(\left(q,c\right),\Lambda_{1}\right),\;S_{\left(q,c\right)}^{\left(q,\cdot \right)}=H\left(\left(q,c\right),\Lambda_{2}\right).$$

It is instructive to apply this to the EPR/Bohm systems A and $A^{‡}$ in (12) and (13). Here, contextuality is traditionally referred to as nonlocality because for the contextual system A, all its couplings are represented in the form of (15): the ineliminable dependence on *c* here is interpreted as the dependence of a measurement on a remote setting. However, if one models the EPR/Bohm experiment by system $A^{‡}$ instead, the HVM representations (16) and (17) both contain the contextualized content $\left(q,c\right)$ as an argument. Following the logic above, they should both be considered nonlocal, even though one of them represents a noncontextual system and is equivalent to (14), while the other represents a contextual system and is equivalent to (15). It seems to us, in agreement with other authors [36], that this demonstration speaks against a naturalistic interpretation of the HVMs in terms of physical dependences.

### 5.3. The Existence and Uniqueness Constraint

In the definition of C-couplings, their reducibility to identity couplings when applied to identically distributed variables is indispensable because without it, the C-contextuality will not be an extension of traditional contextuality. How critical, however, is the second constraint imposed on well-fitting C, that the $C\left[R_{q}\right]$-coupling always exists and is unique? What if one considers statement C for which $C\left[R_{q}\right]$ is a set that may be empty or contain more than one coupling? This complicates the matters conceptually because then, in the consistification procedure, the $\left(q,\cdot \right)$-type bunches, those filled with the $C\left[R_{q}\right]$-couplings, cannot be formed unquely or cannot be formed at all. However, the main point of this paper can still be made, with some qualifications.

We can agree that the consistification of an inconsistently connected system *R* is not a single system $R^{‡}$ but a cluster of systems $\left\{R_{i}^{‡}:i\in I\right\}$, the elements of which are obtained by filling the $\left(q,\cdot \right)$-type bunches in the consistification of *R* by all possible couplings of *R*’s connections. We can further agree that the cluster $\left\{R_{i}^{‡}:i\in I\right\}$ is considered noncontextual if it contains a noncontextual system $R_{i}^{‡}$. In particular, if $\left\{R_{i}^{‡}:i\in I\right\}$ is empty (which means that $C\left[R_{q}\right]$ does not exist for at least one of the connections of *R*), the latter definition is not satisfied, and the cluster should be considered contextual. Once again, we have a theory dealing with consistently connected systems only, except that the empirical or theoretical situations they depict are represented by clusters of systems sharing a format and the $\left(\cdot ,c\right)$-bunches.

It might seem that dealing with an infinity of possible couplings $C\left[R_{q}\right]$ or proving that $C\left[R_{q}\right]$ is empty is a significantly more difficult mathematical task than when C is well-fitting. This is not the case, however, as the complication is not necessarily major. Mathematically, the problem of finding whether a system *R* is contextual consists in determining whether the variable *S* having the same format as *R* can be assigned a probability measure subject to certain constraints on its marginals. The constraints are imposed by the distributions of the bunches $R^{c}$ (that $S^{c}$ have to match) and by the statement C that has to be satisfied by the couplings $S_{q}$ of the connections $R_{q}$. For discrete random variables and finite sets *Q* and *C*, this is a linear programming task, provided that the compliance with C can be presented in terms of linear inequalities of the probabilities in the distribution of $S_{q}$. For the consistification $R^{‡}$ the problem is precisely the same, except that in place of connection couplings, one deals with $\left(q,\cdot \right)$-type bunches.
